# Obesity risk in rural, urban and rural-to-urban migrants: prospective results of the PERU MIGRANT study

**DOI:** 10.1038/ijo.2015.140

**Published:** 2015-08-25

**Authors:** R M Carrillo-Larco, A Bernabé-Ortiz, T D Pillay, R H Gilman, J F Sanchez, J A Poterico, R Quispe, L Smeeth, J J Miranda

**Affiliations:** 1 CRONICAS Center of Excellence in Chronic Diseases, Universidad Peruana Cayetano Heredia, Lima, Peru; 2 Medical School, University College London, London, UK; 3 Department of International Health, Bloomberg School of Public Health, Johns Hopkins University, Baltimore, MD, USA; 4 US Naval Medical Research Unit No. 6 (NAMRU-6), Lima, Peru; 5 Primary Healthcare Center Santa Cruz de Ratacocha, Ministry of Health, Huanuco, Peru; 6 Faculty of Epidemiology and Population Health, London School of Hygiene and Tropical Medicine, London, UK; 7 Department of Medicine, School of Medicine, Universidad Peruana Cayetano Heredia, Lima, Peru

## Abstract

**Background:**

Although migration and urbanization have been linked with higher obesity rates, especially in low-resource settings, prospective information about the magnitude of these effects is lacking. We estimated the risk of obesity and central obesity among rural subjects, rural-to-urban migrants and urban subjects.

**Methods:**

Prospective data from the PERU MIGRANT Study were analyzed. Baseline data were collected in 2007–2008 and participants re-contacted in 2012–2013. At follow-up, outcomes were obesity and central obesity measured by body mass index and waist circumference. At baseline, the primary exposure was demographic group: rural, rural-to-urban migrant and urban. Other exposures included an assets index and educational attainment. Cumulative incidence, incidence ratio (IR) and 95% confidence intervals (95% CI) for obesity and central obesity were estimated with Poisson regression models.

**Results:**

At baseline, mean age (±s.d.) was 47.9 (±12.0) years, and 53.0% were females. Rural subjects comprised 20.2% of the total sample, whereas 59.7% were rural-to-urban migrants and 20.1% were urban dwellers. A total of 3598 and 2174 person-years were analyzed for obesity and central obesity outcomes, respectively. At baseline, the prevalence of obesity and central obesity was 20.0 and 52.5%. In multivariable models, migrant and urban groups had an 8- to 9.5-fold higher IR of obesity compared with the rural group (IR migrants=8.19, 95% CI=2.72–24.67; IR urban=9.51, 95% CI=2.74–33.01). For central obesity, there was a higher IR only among the migrant group (IR=1.95; 95% CI=1.22–3.13). Assets index was associated with a higher IR of central obesity (IR top versus bottom tertile 1.45, 95% CI=1.03–2.06).

**Conclusions:**

Peruvian urban individuals and rural-to-urban migrants show a higher incidence of obesity compared with their rural counterparts. Given the ongoing urbanization occurring in middle-income countries, the rapid development of increased obesity risk by rural-to-urban migrants suggests that measures to reduce obesity should be a priority for this group.

## Introduction

Over the last three decades, the global prevalence of overweight and obesity has increased in both men and women from 28% to 36% and 29% to 38%, respectively.^1, 2^ Decreasing the prevalence of obesity would lower the premature mortality rate due to non-communicable diseases in the general population, and greater benefits would be seen in developing countries.^3^ The obesity-related burden is increasing worldwide, and its association with a number of non-communicable diseases has been described.^4, 5, 6, 7^ A better characterization of the risk patterns of obesity in developing countries, especially those under ongoing processes of urbanization and within-country rural-to-urban migration, remains to be studied.

Most studies report urban and rural-to-urban migrant individuals to be at higher risk of obesity in developing settings.^8, 9, 10, 11^ Possible explanations for urban obesogenic environments relative to rural settings include higher fat and energy intake^12, 13^ and lower levels of physical activity^9, 14, 15^ because of sedentary jobs and passive transportation means.^16^ Provided that these features are different between rural and urban areas in Peru, we expect to see at follow-up different obesity risk profiles among rural, rural-to-urban and urban subjects.^15, 17^

Peru is a developing country undergoing a rapid epidemiological and nutritional transition.^18, 19^ Over the last 30 years, a great proportion of the population has moved from rural to urban areas, primarily because of political violence targeted at rural individuals.^16, 20, 21^ This characteristic offers an ideal scenario in which to assess the risk of rural-to-urban migration in the development of obesity in a context with less selection bias than that introduced by socioeconomic migration. Consequently, we aimed to determine the incidence and risk of general obesity (determined by body mass index, BMI) and central obesity (determined by waist circumference) in rural, rural-to-urban migrant and urban groups. Moreover, we aimed to determine whether assets index or educational attainment is associated with a higher risk of developing general or central obesity, as these two factors are good proxies of socioeconomic status. We hypothesized that higher socioeconomic status would be associated with a higher risk of obesity independently of demographic group.

## Materials and methods

### Study design

This is a prospective cohort study using data of the PERU MIGRANT Study.^16^ The baseline evaluation was conducted in 2007–2008, and aimed to assess the magnitude of differences between rural-to-urban migrant and non-migrant rural and urban groups in relation to specific cardiovascular risk factors.^16^ The follow-up study was conducted in 2012–2013.

### Setting

The study was conducted in urban and rural settings: San Jose de Secce, in the province of Huanta in the Department of Ayacucho, which was considered rural, and ‘Pampas de San Juan de Miraflores' in Lima, which was considered urban. Both urban and rural-to-urban migrant participants were selected from the urban setting.^16^

### Participants

At baseline, eligibility criteria included individuals aged ⩾30 years old permanently living in their place of residence. Initially, the PERU MIGRANT study was conceived as a cross-sectional study to assess the different cardiovascular profiles among rural, rural-to-urban and urban subjects. Although cardiovascular risk factors (that is, obesity, hypertension or diabetes) can be found in younger subjects, these conditions are more common among adults. Being pregnant or having any mental disorder were ineligible to be enrolled. The PERU MIGRANT study followed a single-stage random sampling technique.^16^ At baseline, 20.2% were rural dwellers, 59.7% were rural-to-urban migrants and 20.1% were urban dwellers. Other details about the study groups at baseline have been published elsewhere.^19^

There were 989 individuals at baseline, and 7 were excluded because of missing data in BMI and waist circumference. Follow-up was conducted by contacting participants in the same settings where they were enrolled at baseline. For our present analyses, we included participants with available information on BMI and waist circumference. The follow-up rate at 5 years was 94%: 895 participants were re-assessed and 33 deaths were recorded. For incidence analysis when the outcome was obesity and central obesity, we included 786 and 466 participants, respectively (Supplementary Figure 1).

### Variables

#### Exposure variables

The demographic group (rural, rural-to-urban migrant and urban) as assessed at baseline was the exposure of interest. All subjects were randomly selected from the updated census in the area of residence. Rural subjects included any individuals who permanently lived in San Jose de Secce; migrants included any individuals who reported having been born in Ayacucho but at the time of the study permanently lived in Las Pampas de San Juan de Miraflores; urban dwellers were any individual who reported having been born in Lima and permanently lived in Las Pampas de San Juan de Miraflores. Although some participants could have become migrants between the baseline and follow-up assessment, we did not include such variations in the analysis.

In addition, the assets index and educational attainment as assessed at baseline were also considered exposures of interest. The assets index was constructed as a weighted index based on possession of household assets: gas cooker, radio, black and white television, color television, refrigerator, computer, telephone, cell phone, cable TV, internet, bicycle, motorcycle and car.^22^ This index was divided into tertiles. Educational attainment was defined as no education/incomplete primary education, complete primary education or some secondary education/higher. These variables were collected using standardized questionnaires and procedures as described elsewhere.^16^

#### Outcome variables

The outcomes of general and central obesity were assessed at follow-up. General obesity was defined as BMI ⩾30 kg m^−^^2^, whereas central obesity was defined using waist circumference cutoff points for Ethnic Central and South American populations: ⩾80 cm for women and ⩾90 cm for men.^23^ The anthropometric assessment (BMI and waist circumference) was conducted by trained field workers following standardized procedures.

#### Other variables

We included covariates, all of which were collected at baseline using standardized questionnaires and procedures as described elsewhere.^16^ These included sex (male and female) and age (30–39, 40–49, 50–59 and ⩾60 years). Physical activity was assessed utilizing the International Physical Activity Questionnaire, and categorization of this variable considered total days of physical activity and metabolic equivalents (MET-minutes/week). Moderate physical activity was coded as 5 or more days of any combination of walking, moderate-intensity or vigorous-intensity activities achieving at least 600MET-minutes/week; high physical activity was coded as 7 or more days of any combination of walking, moderate-intensity or vigorous-intensity activities achieving a minimum total physical activity of at least 3000MET-minutes/week. Individuals with low physical activity were those who did not meet the moderate or high physical activity criteria. Smoking was categorized by current smoking status (yes/no); alcohol consumption was categorized by heavy drinking status (yes/no), defined as having a hangover or ⩾6 drinks on the same occasion at least once per month.

### Statistical analysis

The STATA 11.0 statistical package (StataCorp, College Station, TX, USA) was used to conduct the analyses. For descriptive analysis, and to characterize the study population according to the demographic group, we calculated the proportion, mean, standard deviation and 95% confidence intervals (95% CIs) of the outcomes of interest as well as for variables such as age and sex. At baseline, comparisons between categorical variables (all variables described in the variables section were treated as categorical variables) were performed using *χ*^2^ test (for example, we compared the outcomes of interest—obesity and central obesity—versus sex, age or educational attainment). Cumulative incidence of general and central obesity per 100 person-years and 95% CI were calculated, after excluding those participants who had either outcome at baseline, for example, excluding participants who were obese at baseline when using general obesity as the outcome. Incidence ratio (IR) and 95% CI were estimated using generalized linear models with Poisson family and log link, fitting the model using robust standard errors to account for the cluster effect because of the fact that each participant was assessed twice (baseline and follow-up). Regression models were adjusted for several potential confounders including age, sex, physical activity, smoking and alcohol consumption. We developed two regression models: model A was adjusted by sex, age, education and asset index; and model B as in model A plus physical activity, smoking and alcohol consumption.

### Ethics

The baseline assessment was approved by the ethics committees of Universidad Peruana Cayetano Heredia (UPCH) in Peru and the London School of Hygiene and Tropical Medicine in the United Kingdom. The UPCH's Institutional Review Board approved the follow-up in 2012 (SIDISI code 60014). Participants provided written and oral consent at baseline and follow-up, respectively.

## Results

### Population characteristics at baseline

At baseline, the mean age (±s.d.) was 47.9 (±12.0) years, and 53.0% were females. The overall prevalence of obesity was 20.0% (95% CI=17.5–22.5%), and overall prevalence of central obesity was 52.5% (95% CI=49.4–55.7%). The distribution of these outcomes by demographic group is shown in Table 1. The profiles of obesity and central obesity with respect to other variables are shown in Supplementary Tables 1 and 2.

### Incidence and risk of developing general obesity according to demographic group

The mean follow-up time was 5.2 years. When the outcome was general obesity, 3598 person-years were included in the analysis. The cumulative incidence of general obesity was 1.8 (95% CI=1.4–2.3) per 100 person-years. In the rural, migrant and urban groups, the cumulative incidence was 0.4 (95% CI=0.1–1.0), 2.3 (95% CI=1.7–3.1) and 2.6 (95% CI=1.6–4.3), respectively (*P*<0.001). Interestingly, urban women had the highest incidence of general obesity, whereas migrant women had the highest incidence of central obesity. The cumulative incidence of general obesity with respect to other variables is shown in Supplementary Table 3. After adjusting for potential confounders, there was an eightfold higher risk of developing obesity among the migrants and a ninefold higher risk of becoming obese among the urban population, in comparison to the rural group (Table 2).

### Incidence and risk of developing central obesity according to demographic group

For the outcome of central obesity, 2174 person-years were included in the analysis. The cumulative incidence of central obesity was 5.6 (95% CI=4.6–6.6) per 100 person-years: 3.5 (95% CI=2.4–4.9) in the rural group, 7.1 (95% CI=5.7–9.0) in the migrant group and 6.6 (95% CI=4.2–10.4) in the urban group (*P*=0.001). The cumulative incidence of central obesity with respect to other variables is shown in Supplementary Table 3. Taking the rural group as reference and adjusting for potential confounders, there was a higher risk of developing central obesity among the migrant (IR=1.95; 95% CI=1.22–3.13) but not the urban (IR=1.83; 95% CI=0.98–3.41) group (Table 2).

### Risk of developing general obesity or central obesity by educational attainment or wealth index

There was generally no risk of developing general obesity according to assets index or educational attainment (Table 3). However, relative to the bottom tertile, those at the top tertile of the assets index were at higher risk of developing central obesity (IR=1.45; 95% CI=1.03–2.05).

## DISCUSSION

### Main results

Relative to the rural group, there was a substantial 8- to 9.5-fold higher risk of developing general obesity in the migrant and urban groups, and a 95% increased risk of developing central obesity among the migrant population only. Neither assets index nor educational attainment determined a higher risk of becoming obese, although a higher risk of developing central obesity was observed when comparing the top and bottom tertile within the assets index. Within Peru, the magnitude of risk estimates for developing obesity or central obesity were different between residents of rural or urban areas, as well as in those who have moved between these areas. This calls for special attention to the health needs of these demographic groups, focusing on risk factors for non-communicable diseases.

### Demographic group and risk of obesity

Obesogenic environments might explain why people living in urban areas, whether urban dwellers or rural-to-urban migrants, show a high risk of obesity relative to rural subjects. Evidence from cross-sectional and longitudinal studies suggests that migrant and urban individuals are more physically inactive and sedentary relative to rural participants.^9, 14, 15^ Accordingly, we found that the majority of subjects in the low physical activity category were migrants, followed by urban and then rural subjects (data not shown). However, our risk estimates are independent of physical activity status at baseline, so unhealthy physical activity profiles may not fully explain the results. Because obesity results from a misbalance between physical activity and diet, unhealthy diets might be another explanation. Unfortunately, we did not collect data on diet patterns. However, some studies have reported that saturated fat and energy-dense food consumption are higher in urban individuals, followed by migrants and rural individuals.^9, 12^ Although there does not seem to be a prominent difference in fruit and vegetable consumption among these three groups,^8^ urban subjects appear to have a higher consumption of high-energy foods.

Another possible explanation for the findings is that socioeconomic status, combined with access to a Western lifestyle, has an impact on weight. Although rural-to-urban migrants seem to be richer than their rural counterparts,^8^ a wealth index upgrade may not fully explain the different risk estimates: a study from the United States of America showed that among immigrants followed from adolescence to adulthood, individuals with socioeconomic upward mobility had significant lower mean BMI in adulthood.^24^ Based on these findings, it appears that a better socioeconomic position needs to be accompanied by access to an obesogenic environment to result in a higher obesity risk. An obesogenic environment would include several characteristics: improved transit leading to less physical activity related to commuting, a wider access to different kinds of food (healthy, unhealthy and even junk food), wider exposure to fast food and their associated marketing strategies, and different prices between healthy and unhealthy food.^25^ Thus, the rural environment might have a protective effect. Although we did not assess Western lifestyle and environmental characteristics *per se*, our results allow us to hypothesize that these features have indeed a determinant role in the increased risk of obesity among the migrant group.

Contrary to our findings, a Chinese study reported higher obesity incidence in rural versus urban subjects.^26^ Although they defined obesity differently (BMI⩾28.0), their results may suggest that Chinese rural settings are transitioning toward urbanization patterns of obesity faster than Peruvian rural populations. In *post-hoc* analysis, the trend of our incidence estimates was not different from our results when we used the same BMI cutoff as in the Chinese study. There are longitudinal and cross-sectional studies that have reported similar findings to our overall results.^11^ For instance, in Tanzania, an 1-year follow-up of a rural-to-urban migrant cohort reported a mean increase in BMI values,^13^ and cross-sectional studies in India found that rural-to-urban migration status yields higher odds of obesity, relative to rural residents.^8, 9^

### Assets index and educational attainment as risk factors for obesity

In developing countries, there are mixed results regarding the association between wealth index and obesity: some authors report a negative association, others a positive association, whereas others found no significant association.^27^ Regarding assets index, we found a higher risk of developing central obesity among the better-off versus the worse-off. The risk estimates were independent of physical activity, so perhaps dietary patterns are an important determinant. Healthy diets could be more easily affordable by better-off subjects.^25, 28, 29^ Thus, improving the access to or acknowledgment of the importance of healthy diets may be needed among better-off individuals.

We did not find a higher risk of developing general or central obesity at the highest level of educational attainment. Similar results were retrieved in a Hispanic population from the United States, which did not show higher hazards of developing obesity at age 40 years according to educational attainment at age 25 years.^30^ In Spain and China, there seems to be less risk of obesity among the better-educated.^26, 31^ As education could be a target of prevention strategies, further studies on the risk of developing obesity according to educational level are needed.

### Strengths and limitations

This study provides evidence from three well-defined groups included in a prospective cohort. Moreover, the prospective design of the study rules out reverse causation. Finally, the study sample was assessed with the same instruments, standardized procedures and in some cases by the same field workers, minimizing the risk for non-differential misclassification bias.

However, limitations should be highlighted. First, although the sampling technique could have minimized, the risk for non-random sampling bias, at baseline the response rate was higher in the rural versus urban group, and among the latter, non-responders and responders differed in educational attainment.^16^ Therefore, when the exposure was educational attainment the results should be taken with caution, as they may not represent the whole variation of educational level among the urban group. Second, we did not have data on diet patterns, and the implications of this limitation have been discussed. This gap is to be filled by future studies, which could determine whether diet patterns or physical activity are more or equally important as obesity determinants across these population groups. Third, attrition bias could also be an issue, although we were able to re-contact most of the participants. Participants who were excluded due to missing values comprised less than 1% of the study population, reducing non-response bias. Finally, the small sample size could have prevented us from finding significant results when assessing the outcomes of interest and the exposure was socioeconomic status.

## Conclusions

In comparison with rural habitants, urban individuals and rural-to-urban migrants have a higher incidence of obesity, and only migrants were at higher risk of central obesity. After adjusting for demographic group, educational attainment or assets index did not determine higher risk of obesity. Given the urbanization occurring in many middle-income countries, the rapid development by rural-to-urban migrants of increased obesity risks seen in long-term urban dwellers suggests that measures to reduce obesity should be a priority for migrants into urban areas.

## Figures and Tables

**Table 1:** Distribution of general and central obesity by demographic group at baseline. The PERU MIGRANT study

	*General obesity (%)*		P*-value*	*Central obesity (%)*		P*-value*
	*No (*n=*786)*	*Yes (*n=*196)*		*No (*n=*466)*	*Yes (*n=*516)*	
Rural	97.5	2.5	<0.001	84.9	15.2	<0.001
Migrant	78.8	21.2		39.6	60.4	
Urban	66.2	33.8		33.3	66.7	

**Table 2:** Relative risk of general obesity (BMI) or central obesity (waist circumference) by demographic group. The PERU MIGRANT study

	*Crude model*	*Model A*	*Model B*
	*IR (95% CI)*	*IR (95% CI)*	*IR (95% CI)*
*Outcome: general obesity (*n=*680)*			
Rural	1	1	1
Migrant	**6.01 (2.20–16.44)**	**6.36 (2.19–18.47)**	**8.19 (2.72–24.67)**
Urban	**6.74 (2.29–19.80)**	**7.13 (2.16–23.55)**	**9.51 (2.74–33.01)**
*Outcome: central obesity (*n=*386)*			
Rural	1	1	1
Migrant	**1.96 (1.36–2.82)**	**2.15 (1.40–3.29)**	**1.95 (1.22–3.13)**
Urban	**1.75 (1.07–2.86)**	**2.03 (1.14–3.62)**	1.83 (0.98–3.41)

Abbreviations: BMI, body mass index; CI, confidence interval; IR, incidence ratio.

Model A: sex, age, education, and asset index; all assessed at baseline. Model B: model A plus physical activity, smoking and alcohol consumption; all assessed at baseline. In bold, *P*<0.05.

**Table 3:** Relative risk of general obesity and central obesity according to sociodemographic variables. The PERU MIGRANT study

	*Crude*	*Adjusted*	*Crude*	*Adjusted*
	*Outcome: general obesity (*n=*680)*		*Outcome: central obesity (*n=*386)*	
	*IR (95% CI)*	*IR (95% CI)*	*IR (95% CI)*	*IR (95% CI)*
*Assets index*				
Bottom	1	1^a^	1	1^a^
Middle	1.43 (0.82–2.50)	0.97 (0.54–1.76)	1.33 (0.88–2.02)	1.09 (0.70–1.69)
Top	0.92 (0.52–1.62)	0.79 (0.45–1.39)	**1.50 (1.07–2.10)**	**1.45 (1.03–2.05)**
*Education*				
Non/some primary	1	1^b^	1	1^b^
Complete primary	1.40 (0.63–3.10)	0.87 (0.40–1.90)	1.26 (0.77–2.06)	1.00 (0.59–1.70)
Secondary or higher	**1.80 (1.02–3.18)**	0.87 (0.45–1.66)	1.32 (0.93–1.86)	0.80 (0.51–1.25)

Abbreviations: CI, confidence interval; IR, incidence ratio.

a Adjusted by education, sex, age, demographic group, physical activity, smoking and alcohol consumption; all at baseline.

b Adjusted by assets index, sex, age, demographic group, physical activity, smoking and alcohol consumption; all at baseline.

b In bold, *P*<0.05.
