# Supplementary material for: Obesity risk in rural, urban and rural-to-urban migrants: prospective results of the PERU MIGRANT study
Source: Int J Obes (Lond). 2015 Aug 25;40(1):181–5. doi: 10.1038/ijo.2015.140 (PMC4677453; doi:10.1038/ijo.2015.140)
Supplement: Supplementary Figure 1 [file ijo2015140x1.docx]

## Supplementary Figure 1: Flowchart of the participants included in the prospective analysis. The PERU MIGRAN Study.

Excluded because of missing values (BMI or waist circumference) at baseline (n=7)

Analysis of cumulative incidence and risk of central obesity (n=466)

Excluded because had central obesity at baseline (n=516)

Excluded because had obesity at baseline (n=196)

Analysis of cumulative incidence and risk of obesity (n=786)

PERU MIGRANT (n=989)

Participants at baseline (n=982)
