# Supplementary material for: Obesity risk in rural, urban and rural-to-urban migrants: prospective results of the PERU MIGRANT study
Source: Int J Obes (Lond). 2015 Aug 25;40(1):181–5. doi: 10.1038/ijo.2015.140 (PMC4677453; doi:10.1038/ijo.2015.140)
Supplement: Supplementary Table 1 [file ijo2015140x2.docx]

## Supplementary Table 1: Distribution of sociodemographic and risk factors according to obesity (BMI) and by demographic group at baseline. The PERU MIGRANT study.

| Variable | Rural | | | Migrant | | | Urban | | |
| --- | --- | --- | --- | --- | --- | --- | --- | --- | --- |
|  | No | Yes | P | No | Yes | P | No | Yes | p |
| Sex | n=193 | n=5 | 0.031 | n=462 | n=124 | <0.001 | n=131 | n=67 | 0.001 |
| Female | 51.3 | 100.0 |  | 46.5 | 75.8 |  | 45.0 | 71.6 |  |
| Male | 48.7 | 00.0 |  | 53.5 | 24.2 |  | 55.0 | 28.4 |  |
| Age | n=193 | n=5 | 0.705 | n=462 | n=124 | 0.009 | n=131 | n=67 | 0.038 |
| 30-30y | 30.1 | 40.0 |  | 30.3 | 19.4 |  | 35.1 | 16.4 |  |
| 40-49y | 28.0 | 40.0 |  | 26.6 | 39.5 |  | 24.4 | 31.3 |  |
| 50-59y | 22.3 | 20.0 |  | 27.9 | 30.7 |  | 25.2 | 37.3 |  |
| 60+y | 19.7 | 00.0 |  | 15.2 | 10.5 |  | 15.3 | 14.9 |  |
| Education | n=193 | n=5 | 0.951 | n=461 | n=124 | 0.001 | n=130 | n=67 | 0.055 |
| Non/Some Primary | 65.3 | 60.0 |  | 27.8 | 42.7 |  | 4.6 | 10.5 |  |
| Complete primary | 15.0 | 20.0 |  | 16.3 | 19.4 |  | 8.5 | 16.4 |  |
| Secondary/Higher | 19.7 | 20.0 |  | 56.0 | 37.9 |  | 86.9 | 73.1 |  |
| Assets Index | n=193 | n=5 | 0.429 | n=462 | n=124 | 0.141 | n=131 | n=67 | 0.559 |
| Lowest | 61.7 | 40.0 |  | 41.6 | 39.5 |  | 31.3 | 38.8 |  |
| Middle | 6.7 | 20.0 |  | 24.7 | 33.1 |  | 36.6 | 31.3 |  |
| Highest | 31.6 | 40.0 |  | 33.8 | 27.4 |  | 32.1 | 29.9 |  |
| Physical Activity | n=193 | n=5 | 0.835 | n=456 | n=123 | 0.331 | n=131 | n=66 | 0.033 |
| Low | 2.1 | 0.0 |  | 28.1 | 35.0 |  | 41.2 | 34.9 |  |
| Moderate | 4.7 | 0.0 |  | 37.1 | 33.3 |  | 27.5 | 45.5 |  |
| High | 93.3 | 100.0 |  | 34.9 | 31.7 |  | 31.3 | 19.7 |  |
| Heavy Drinker | n=193 | n=5 | 0.615 | n=462 | n=123 | 0.954 | n=131 | n=67 | 0.033 |
| No | 87.6 | 80.0 |  | 91.8 | 91.9 |  | 87.8 | 97.0 |  |
| Yes | 12.4 | 20.0 |  | 8.2 | 8.1 |  | 12.2 | 3.0 |  |
| Current Smoker* | n=193 | n=5 | 0.583 | n=462 | n=124 | 0.404 | n=131 | n=67 | 0.357 |
| No | 94.3 | 100.0 |  | 89.4 | 91.9 |  | 81.7 | 76.1 |  |
| Yes | 5.7 | 0.0 |  | 10.6 | 8.1 |  | 18.3 | 23.9 |  |

Percentages are presented; p-value for χ2 test.*Assessed at baseline only.
