# Supplementary material for: Obesity risk in rural, urban and rural-to-urban migrants: prospective results of the PERU MIGRANT study
Source: Int J Obes (Lond). 2015 Aug 25;40(1):181–5. doi: 10.1038/ijo.2015.140 (PMC4677453; doi:10.1038/ijo.2015.140)
Supplement: Supplementary Table 2 [file ijo2015140x3.docx]

## Supplementary Table 2: Distribution of sociodemographic and risk factors according to central obesity (waist circumference) and by demographic group at baseline. The PERU MIGRANT study.

| Variable | Rural | | | Migrant | | | Urban | | |
| --- | --- | --- | --- | --- | --- | --- | --- | --- | --- |
|  | No | Yes | P | No | Yes | P | No | Yes | P |
| Sex | n=168 | n=30 | <0.001 | n=232 | n=354 | <0.001 | n=66 | n=132 | <0.001 |
| Female | 45.8 | 90.0 |  | 31.0 | 67.0 |  | 33.3 | 64.4 |  |
| Male | 54.2 | 10.0 |  | 69.0 | 33.1 |  | 66.7 | 35.6 |  |
| Age | n=168 | n=30 | 0.026 | n=232 | n=354 | <0.001 | n=66 | n=132 | 0.019 |
| 30-30y | 26.8 | 50.0 |  | 37.5 | 21.8 |  | 36.4 | 25.0 |  |
| 40-49y | 28.6 | 26.7 |  | 26.7 | 31.1 |  | 21.1 | 30.0 |  |
| 50-59y | 22.6 | 20.0 |  | 22.8 | 32.2 |  | 19.7 | 34.1 |  |
| 60+y | 22.0 | 3.3 |  | 12.9 | 15.0 |  | 22.7 | 11.4 |  |
| Education | n=168 | n=30 | 0.964 | n=232 | n=353 | <0.001 | n=66 | n=132 | 0.581 |
| Non/Some Primary | 65.5 | 63.3 |  | 20.7 | 37.7 |  | 4.6 | 7.6 |  |
| Complete primary | 14.9 | 16.7 |  | 12.9 | 20.0 |  | 9.2 | 12.1 |  |
| Secondary/Higher | 19.6 | 20.0 |  | 66.4 | 42.8 |  | 88.2 | 80.3 |  |
| Assets Index | n=168 | n=30 | 0.230 | n=232 | n=354 | 0.271 | n=66 | n=132 | 0.468 |
| Lowest | 63.1 | 50.0 |  | 42.7 | 40.1 |  | 37.9 | 31.8 |  |
| Middle | 6.0 | 13.3 |  | 22.8 | 28.8 |  | 36.4 | 34.1 |  |
| Highest | 31.0 | 36.7 |  | 34.5 | 31.1 |  | 25.8 | 34.1 |  |
| Physical Activity | n=168 | n=30 | 0.812 | n=227 | n=352 | 0.626 | n=66 | n=131 | 0.403 |
| Low | 1.8 | 3.3 |  | 28.6 | 30.1 |  | 43.9 | 36.6 |  |
| Moderate | 4.8 | 3.3 |  | 34.8 | 37.2 |  | 23.3 | 36.6 |  |
| High | 93.5 | 93.3 |  | 36.6 | 32.7 |  | 28.8 | 26.7 |  |
| Heavy Drinker | n=168 | n=30 | 0.096 | n=232 | n=354 | 0.218 | n=66 | n=132 | 0.009 |
| No | 85.7 | 96.7 |  | 90.1 | 92.9 |  | 83.3 | 94.7 |  |
| Yes | 14.3 | 3.3 |  | 9.9 | 7.1 |  | 16.7 | 5.3 |  |
| Current Smoker* | n=168 | n=30 | 0.773 | n=232 | n=354 | <0.001 | n=66 | n=132 | 0.802 |
| No | 94.6 | 93.3 |  | 83.6 | 94.1 |  | 78.8 | 80.3 |  |
| Yes | 5.4 | 6.7 |  | 16.4 | 5.9 |  | 21.2 | 19.7 |  |

Percentages are presented; p-value for χ2 test. *Assessed at baseline only.
