# Supplementary material for: Obesity risk in rural, urban and rural-to-urban migrants: prospective results of the PERU MIGRANT study
Source: Int J Obes (Lond). 2015 Aug 25;40(1):181–5. doi: 10.1038/ijo.2015.140 (PMC4677453; doi:10.1038/ijo.2015.140)
Supplement: Supplementary Table 3 [file ijo2015140x4.docx]

## Supplementary Table 3: Cumulative incidence of general and central obesity according to sociodemographic variables and risk factors, by study group. The PERU MIGRANT study.

| Variables | General Obesity (Incidence (95%CI)) | | | Central Obesity (Incidence (95%CI)) | | |
| --- | --- | --- | --- | --- | --- | --- |
|  | Rural | Migrant | Urban | Rural | Migrant | Urban |
| Sex |  |  |  |  |  |  |
| Female | 0.5 (0.2-1.7) | 2.2 (1.5-3.4) | 3.1 (1.5-6.2) | 4.4 (2.8-6.9) | 8.0 (5.3-11.9) | 7.0 (3.1-15.5) |
| Male | 0.2 (0.0-1.5) | 2.4 (1.6-3.5) | 2.2 (1.1-4.6) | 2.7 (1.5-4.7) | 6.9 (5.2-9.1) | 6.1 (3.5-10.8) |
| Age |  |  |  |  |  |  |
| 30-30y | 0.6 (0.2-2.5) | 2.4 (1.4-4.0) | 3.3 (1.6-7.0) | 4.0 (2.1-7.4) | 6.9 (4.7-10.2) | 9.2 (4.9-17.1) |
| 40-49y | 0.0 (0.0-0.0) | 2.9 (1.7-4.7) | 2.7 (1.0-7.3) | 4.4 (2.4-7.9) | 6.5 (4.1-10.4) | 3.1 (0.8-12.5) |
| 50-59y | 0.4 (0.1-3.0) | 2.3 (1.3-4.0) | 2.4 (0.9-6.4) | 2.9 (1.3-6.4) | 7.7 (4.9-12.3) | 7.7 (3.2-18.5) |
| 60+y | 0.6 (0.1-4.1) | 1.1 (0.4-3.5) | 0.0 (0.0-0.0) | 2.4 (0.9-6.3) | 8.7 (4.7-16.2) | 2.3 (0.3-16.1) |
| Education |  |  |  |  |  |  |
| None/Some Primary | 0.5 (0.1-1.4) | 2.3 (1.3-4.0) | 0.0 (0.0-0.0) | 3.0 (1.9-4.8) | 9.4 (5.9-14.9) | 10.0 (1.4-71.0) |
| Complete primary | 0.0 (0.0-0.0) | 2.8 (1.5-5.4) | 0.0 (0.0-0.0) | 3.6 (1.5-8.7) | 8.0 (4.3-14.9) | 4.0 (0.6-28.4) |
| Secondary or higher | 0.5 (0.1-3.5) | 2.2 (1.5-3.23) | 3.0 (1.8-4.9) | 5.2 (2.7-9.9) | 6.4 (4.8-8.7) | 6.6 (4.1-10.8) |
| Assets Index |  |  |  |  |  |  |
| Lowest | 0.3 (0.1-1.3) | 2.2 (1.4-3.5) | 4.4 (2.1-9.2) | 2.9 (1.8-4.8) | 7.0 (2.9-10.1) | 4.6 (1.7-12.3) |
| Middle | 1.5 (0.5-10.8) | 3.1 (1.9-5.1) | 1.4 (0.5-4.3) | 3.7 (0.9-14.8) | 6.1 (3.5-10.5) | 7.2 (3.6-14.4) |
| Highest | 0.3 (0.0-2.1) | 1.9 (1.1-3.3) | 2.5 (1.0-6.0) | 4.6 (2.7-7.9) | 8.1 (5.7-11.7) | 7.1 (3.2-15.9) |
| Physical Activity |  |  |  |  |  |  |
| Low | 0.0 (0.0-0.0) | 2.2 (1.2-3.8) | 2.1 (0.9-5.0) | 0.0 (0.0-0.0) | 6.7 (4.2-10.6) | 6.4 (3.2-12.8) |
| Moderate | 0.0 (0.0-0.0) | 2.1 (1.3-3.5) | 1.9 (0.6-5.8) | 5.6 (1.4-22.2) | 8.6 (5.9-12.5) | 7.5 (3.4-16.7) |
| High | 0.4 (0.2-1.1) | 2.8 (1.8-4.3) | 4.0 (1.9-8.5) | 3.5 (2.4-5.0) | 6.6 (4.4-9.8) | 5.2 (1.9-13.8) |
| Heavy Drinker |  |  |  |  |  |  |
| No | 0.4 (0.2-1.2) | 2.2 (1.6-3.0) | 2.9 (1.8-4.9) | 3.5 (2.4-5.2) | 7.3 (5.7-9.3) | 5.9 (3.5-9.9) |
| Yes | 0.0 (0.0-0.0) | 3.4 (1.4-8.2) | 0.0 (0.0-0.0) | 3.3 (1.3-8.9) | 6.2 (2.6-15.0) | 9.1 (3.4-24.2) |
| Current Smoker* |  |  |  |  |  |  |
| No | 0.4 (0.2-1.1) | 2.4 (1.8-3.2) | 2.6 (1.5-4.6) | 3.6 (2.5-5.1) | 7.0 (5.4-9.0) | 6.6 (3.9-11.1) |
| Yes | 0.0 (0.0-0.0) | 1.6 (0.5-4.8) | 2.6 (0.8-8.0) | 2.1 (0.3-14.8) | 8.7 (4.9-15.3) | 5.8 (2.2-15.4) |
